# The relationship between esports and cognitive function: A scoping review

**DOI:** 10.1371/journal.pone.0352875

**Published:** 2026-07-10

**Authors:** Mingyang Lu, Hwanho Lee, Inae Yoon

**Affiliations:** 1 Department of Physical Education, Dankook University, Yongin, South Korea; 2 Graduate School of Education (Department of Education), Dankook University, Yongin-si, South Korea; İzmir Democracy University: Izmir Demokrasi Universitesi, TÜRKIYE

## Abstract

The aim of this scoping review was to map and synthesize empirical evidence on the relationship between esports participation and cognitive function, with particular attention to executive control, attentional processes, visuospatial working memory, decision-making, and lifestyle-related factors. Searches were conducted in PubMed and Web of Science following PRISMA-ScR guidance. Nine empirical studies met the inclusion criteria and were included in the final synthesis. The included evidence consisted mainly of cross-sectional comparisons, together with a small number of intervention, acute experimental, and qualitative studies. The studies were organized into three thematic domains: cognitive differences across expertise levels, lifestyle and intervention effects on cognitive performance, and decision-making or training mechanisms. Five studies primarily examined expertise-level differences, three addressed lifestyle, health load, or intervention-related factors, and one focused on decision-making and training processes. Overall, the available evidence suggests that competitive esports players may show advantages in selected cognitive domains, particularly executive control, attentional flexibility, and visuospatial processing. However, findings were heterogeneous across game genres, participant classifications, cognitive tasks, and reporting practices. Because only nine studies were eligible and most designs were cross-sectional, the current evidence base remains insufficient for strong causal conclusions about whether esports participation improves cognitive function or whether pre-existing cognitive abilities contribute to esports expertise. Future research should use longitudinal, experimental, and cross-cultural designs with standardized cognitive measures and transparent reporting of statistical results.

## Introduction

In recent years, esports has become one of the fastest-growing sports and cultural phenomena worldwide. Popular titles such as League of Legends (LOL), Counter-Strike: Global Offensive (CSGO), Honor of Kings and Dota 2 are structured with ranking systems. The esports industry has expanded rapidly over the past two decades, with continued growth in global audience size and market value [1,2]. The global esports audience reached approximately 640 million in 2025 [1], with further expansion projected in the coming years [2]. This development reflects not only the increasing popularity of esports as a form of digital entertainment but also its transformation into a highly organized and professional competitive industry, providing valuable opportunities for investigating expertise in high-level esports players.

Hamari and Sjöblom [3] define esports as a type of sporting activity facilitated by electronic systems. Its core characteristic lies in achieving competitive performance through interaction with electronic systems within structured gaming environments [4–6]. Competitive esports contexts require rapid perception, sustained attention, working memory, decision-making, and action execution under time pressure [7–9]. Studies comparing different game genres suggest that cognitive demands may vary by title. Action-based games, particularly first-person shooters, often emphasize speeded responses and attentional processing, whereas strategy-oriented or team-based games may place greater demands on planning, cognitive flexibility, and coordination [7,10,11]. Neuroimaging research has reported associations between gaming or esports experience and brain function or structure [12], but such evidence should be interpreted as correlational unless supported by longitudinal or experimental designs. Accordingly, esports may provide an applied context for mapping task-specific cognitive demands, rather than direct evidence that esports participation causes broad cognitive or neural adaptation. Cognitive function refers to the mental processes involved in acquiring knowledge, manipulating information, and reasoning [13]. In esports performance, these processes are particularly relevant to attentional control, working memory, and decision-making under time pressure [9]. Prior studies and reviews suggest that esports and related action video-game contexts may be associated with selected cognitive differences, including attentional control, visuospatial processing, and rapid information processing [7,14,15]. Neuroimaging evidence has also reported associations between esports or video-game experience and brain function or structure [12], but such findings should be interpreted as correlational unless supported by longitudinal or experimental evidence. Research on esports structure and training further indicates that competitive status, game genre, and performance context should be considered when interpreting cognitive findings [16,17]. In addition, emerging studies suggest that physical exercise and lifestyle factors may be relevant to cognitive performance in esports players [18,19], but this evidence remains limited and heterogeneous.

Nevertheless, several important gaps remain in the current literature. First, many existing studies and reviews have focused on recreational gamers or mixed gaming populations rather than clearly defined competitive esports players, which limits the specificity of conclusions regarding expertise-related cognitive performance. Second, although previous reviews have examined psychological, physiological, or health-related aspects of esports participation, relatively few have synthesized empirical evidence specifically targeting cognitive function in competitive esports players. Third, prior studies have often examined isolated outcomes rather than providing an integrated synthesis of key cognitive domains—such as executive control, attentional processes, and visuospatial working memory—together with lifestyle- and training-related factors that may shape performance in esports contexts.

Given these limitations, a scoping review represents an appropriate methodological approach for this emerging and interdisciplinary field because it allows existing evidence to be systematically mapped, conceptually organized, and examined for gaps across heterogeneous study designs [20]. Accordingly, the present review focuses specifically on competitive esports players and aims to provide a structured synthesis of cognitive function in esports contexts. More specifically, this review has three objectives: (1) to synthesize evidence on differences in cognitive function across levels of esports expertise; (2) to examine the effects of lifestyle factors, health load, and intervention strategies on cognitive and performance outcomes; and (3) to explore the decision-making and training mechanisms underlying high-level esports performance. By addressing these objectives, the present review seeks to clarify the cognitive characteristics associated with esports expertise and to identify key research patterns and remaining gaps in this rapidly developing area.

## Materials and methods

### Data source and search strategy

As previously noted, research on the relationship between esports and cognitive function remains relatively underexplored [9]. Consequently, there is still limited consensus regarding which specific variables most strongly influence cognitive function. Investigations into these variables span a broad and multidisciplinary field, drawing insights from engineering, computer science, psychology, and human factors research. Relevant determinants may include game design characteristics, ergonomic features, physical and psychological components, as well as the unique attributes of individual game genres [17]. Given this diversity, the volume and scope of related studies are extensive. Over time, as research methodologies mature, these factors are expected to be more clearly delineated, enabling more focused empirical investigation [9]. Recognizing the evolving nature of this field, the present study adopts a scoping review approach as a first step toward a structured, data-driven contribution to the expanding body of esports research. This method provides a comprehensive foundation for mapping existing evidence and identifying critical pathways for future scientific inquiry into the cognitive dimensions of competitive gaming.

The present review follows the Preferred Reporting Items for Systematic Reviews and Meta-Analyses Extension for Scoping Reviews (PRISMA-ScR) guidelines [21]. A protocol record was made available on the Open Science Framework (OSF; https://osf.io/r2ckv/; DOI: 10.17605/OSF.IO/R2CKV) to enhance transparency in the review objectives, eligibility criteria, planned data extraction procedures, and synthesis strategy.

Two independent reviewers (LMY and LHH) conducted systematic searches between March and May 2025 in two primary electronic databases: PubMed and Web of Science. These databases were selected because they provide broad coverage of peer-reviewed literature across psychology, neuroscience, medicine, sport science, and interdisciplinary esports-related research. PubMed and Web of Science were used for primary systematic retrieval and screening, whereas ScienceDirect was used only as a supplementary source for full-text access, reference checking, and manual verification rather than as a primary screening database.

The PubMed search yielded 153 records and used the following search string: ((Esport) OR (Esports) OR (e-sport) OR (e-sports) OR (electronic game) OR (online game)) AND ((cognitive function[Title]) OR (cognition[Title]) OR (executive function[Title]) OR (inhibition[Title]) OR (attention[Title]) OR (working memory[Title]) OR (shifting[Title])). The Web of Science search yielded 2,907 records and used the following search string: TS=(esport OR esports OR “e-sport” OR “e-sports” OR “electronic gam*” OR “online gam*”) AND TS=(“cognitive function*” OR cognition* OR “executive function*” OR inhibition* OR attention* OR “working memory*” OR shifting). The search was limited to peer-reviewed English-language publications. The search terms were deliberately broad to capture studies addressing esports participation, competitive gaming, and cognitive-function outcomes, while the subsequent eligibility assessment was used to determine whether each record matched the target population and conceptual scope of this review.

### Selection of studies: Inclusion and exclusion criteria

Given the objectives of this scoping review, a broad inclusion strategy was adopted. Empirical studies were considered eligible if they employed experimental designs (e.g., randomized controlled trials, laboratory-based interventions), observational approaches (cross-sectional, longitudinal, or case–control studies), or qualitative/mixed-method designs. Non-empirical publications—such as narrative reviews, commentaries, editorials, and conference abstracts—were excluded because they do not provide directly comparable data. The inclusion of multiple research designs was justified by the developmental stage of esports–cognition research. Experimental studies offer insights into causal mechanisms and short-term effects (e.g., training or exercise interventions), whereas observational studies capture long-term trends and group-level characteristics (e.g., cognitive profiles across expertise levels). In addition, qualitative and mixed-method designs contribute valuable interpretive perspectives from players and coaches. Accordingly, for the purposes of this scoping review, studies were included if they met the following criteria: a) the article reported original research; b) the study focused on esports or competitive video gaming; c) the publication was written in English and appeared in a peer-reviewed journal; d) the study content was directly related to cognitive function. The exclusion criteria were as follows: a) studies focusing exclusively on casual or recreational gaming rather than competitive esports; b) studies whose participants comprised general populations rather than professional, semi-professional, or academy-level esports players; c) publications inconsistent with the purpose of this review or not meeting the above inclusion criteria, including related reviews or systematic reviews (with or without meta-analyses). Studies were excluded if they met any of the following criteria: (1) the sample consisted only of recreational or casual video game players without a competitive esports context; (2) the study did not report cognitive-function outcomes or did not provide extractable cognitive-performance data; (3) the publication was a review, editorial, conference abstract, commentary, thesis, or other non-empirical source; (4) the study focused on general video gaming, gaming disorder, or psychological outcomes without a direct cognitive-function component; (5) the study did not provide sufficient methodological or outcome information for extraction; or (6) the article was not published in English. Studies were not excluded on the basis of whether their findings were statistically significant.

### Data extraction and data management

Two reviewers (LMY and LHH) independently conducted literature searches using the predefined search terms across the selected meta-search engines (PubMed and Web of Science). Following the database search, all retrieved records were exported and screened for eligibility. Titles and abstracts were independently reviewed by two researchers based on predefined inclusion and exclusion criteria. Full-text articles were then assessed to determine final eligibility. ScienceDirect was used only as a supplementary source for cross-checking references and identifying additional potentially relevant studies, rather than as part of the primary screening process. In the next stage, the full texts of the preliminarily selected articles were examined to determine their final eligibility for inclusion in the scoping review.

Any disagreements arising during the inclusion process were resolved through discussion and consensus. If consensus could not be reached, a third reviewer facilitated a final adjudication meeting to reach a resolution.

The study selection process is illustrated in S1 Fig. A total of 3,060 records were identified from the two primary databases: 2,907 from Web of Science and 153 from PubMed. Before title and abstract screening, 41 records were removed, including 23 duplicate records and 18 records removed during database-record cleaning. Therefore, 3,019 records were screened by title and abstract. Of these, 2,984 records were excluded because they were clearly unrelated to esports and cognitive function, did not involve relevant empirical outcomes, or did not match the conceptual scope of the review. The remaining 35 reports were assessed for eligibility. After full eligibility assessment, 26 reports were excluded because they did not involve competitive esports populations, focused only on recreational gaming or non-esports video game contexts, did not report extractable cognitive-function outcomes, were non-empirical publications, or lacked sufficient methodological information for data extraction. Nine studies met all inclusion criteria and were included in the final synthesis.

A standardized data extraction form was used to ensure consistency across studies. The following information was extracted from each included study: author(s), year of publication, country or region, esports title or game genre, participant characteristics, competitive status, study design, cognitive domain examined, cognitive task or measurement instrument, main quantitative findings, reported p values or effect sizes where available, and authors’ main conclusions. When a study did not report a p value, effect size, or confidence interval for a specific finding, this was recorded as “NR” rather than estimated post hoc. Extracted data were checked independently by two reviewers, and disagreements were resolved through discussion. This procedure was used to improve transparency and to avoid selective reporting of only statistically significant findings.

### Assessment of the quality of studies

To appraise the methodological reporting quality of the included studies, two reviewers (LMY and YIA) applied the Joanna Briggs Institute (JBI) Critical Appraisal Checklist [22]. Because this review was designed as a scoping review, the appraisal was used to describe the methodological transparency and reporting quality of the evidence base rather than to exclude studies or to generate a formal risk-of-bias rating. The appraisal focused on whether each study reported clear eligibility criteria, participant characteristics, measurement procedures, outcome definitions, and appropriate statistical analyses. Disagreements were resolved through discussion. The results of the appraisal are presented in S1 Table and are interpreted descriptively rather than as a basis for excluding studies from the review.

## Results

This section synthesizes the findings of the nine included studies, as summarized in S2 Table. Given the substantial heterogeneity in study design, esports title or genre, participant classification, cognitive tasks, and statistical reporting, the findings were summarized narratively rather than pooled quantitatively. The synthesis was organized into three thematic domains: (1) cognitive function and expertise-level differences, (2) lifestyle, health load, and intervention effects, and (3) decision-making and training mechanisms. This structure was used to compare the direction and consistency of findings across studies while avoiding selective emphasis on isolated statistically significant results. Reported p values, effect sizes, or confidence intervals are presented in S2 Table where available; when such statistics were not reported in the original articles, the relevant entry was recorded as “NR”.

### Cognitive function and expertise-level differences

Five studies primarily examined cognitive function in relation to esports expertise level, player classification, or game genre. Across these studies, participants included professional players, semi-professional or university-level players, long-term players from FPS or MOBA genres, and comparison groups. The study designs were mainly cross-sectional or comparative, with some studies using neuropsychological testing, computerized cognitive tasks, or neuroimaging-related measures. The cognitive domains examined included sustained attention, inhibitory control, reaction-time performance, visuospatial working memory, attentional control, and sensorimotor coordination. Overall, the evidence suggests domain-specific cognitive differences associated with expertise level or game genre rather than a uniform cognitive advantage across all tasks. Findings appeared more consistent for attention, visual-attentional control, and speeded or sensorimotor processing, whereas evidence for working memory and inhibitory control was more mixed across studies.

In a neuropsychological study involving 14 professional Overwatch players, standardized testing revealed superior performance in sustained attention (Cohen’s d ≈ 0.84) and visual memory (d ≈ 1.02), but slightly lower scores in spatial working memory (d ≈ 0.79). Players also exhibited faster reaction times but higher error rates on response-time tasks (p < 0.05) [23]. Additionally, some studies have reported that esports players share similar psychological characteristics with traditional athletes, while also exhibiting distinct cognitive differences, particularly in working memory and emotional processing [24].

In a Korean KeSPA professional-league sample (N = 55), players were classified as elite (season participation ≥ 80%, n = 12) or general professional (n = 43). The elite group demonstrated significantly better planning ability (ToL reaction time = 45.6 ± 8.7 vs. 62.1 ± 11.3, p < 0.01) and spatial representation accuracy (MR accuracy = 82% vs. 69%), along with lower state anxiety (p < 0.01) [4]. These findings indicate that cognitive performance remains associated with competitive standing even within professional tiers.

A comparative study of esports players showed that FPS players had higher correct responses and faster reaction times in attention-related tasks, whereas MOBA players demonstrated relatively different cognitive performance patterns across tasks (p < 0.05) [11].

In a League of Legends expert sample (N = 66), experts scored significantly higher in spatial working memory and attentional control than semi-professionals and non-experts (p < 0.01), with similar patterns also reported in comparisons between expert, regular, and non-player groups [7,14,25]. Collectively, these studies suggest that esports players may exhibit advantages in specific cognitive domains compared to non-player or lower-expertise groups. Differences in cognitive performance also appear to vary across expertise levels and game genres. However, given the limited number of studies and the heterogeneity of study designs, these findings should be interpreted with caution. Additional evidence from neuroimaging and behavioral studies indicates that long-term esports players may exhibit enhanced visual attention and sensorimotor processing abilities [26].

### Lifestyle, health load, and intervention effects

This thematic domain included three studies that examined acute exercise, competitive match load, and lifestyle-related factors in relation to cognitive performance. The evidence was heterogeneous in design and measurement approach, including one randomized crossover exercise trial, one pre–post observational assessment of a competitive gaming session, and one cross-sectional nutrition and lifestyle study. Therefore, the findings were interpreted as preliminary evidence that health-related and physiological factors may be associated with selected cognitive outcomes in esports players rather than as definitive evidence for a causal lifestyle–cognition pathway.

Zhang et al. [27] examined the effects of acute moderate-intensity aerobic exercise in 34 E-athletes using a randomized crossover design. Participants completed 30 minutes of power cycling at 64–76% of maximum heart rate, and cognitive outcomes were assessed before, immediately after, and 30 minutes after exercise using Human Benchmark tasks. The exercise condition was associated with immediate improvements in speed accuracy, visual memory, and reaction time, with reported effects including speed accuracy (P < .001, Cohen’s d = 1.406, 95% CI: 0.717–2.072), visual memory (P < .001, Cohen’s d = 1.416, 95% CI: 0.725–2.086), and reaction time (P < .001, Cohen’s d = 1.265, 95% CI: 0.610–1.898). Some effects, particularly for speed accuracy and reaction time, were reported to persist 30 minutes after exercise, whereas sequence memory and the chimp test did not show significant changes. These findings suggest that acute aerobic exercise may transiently support selected speeded and visual cognitive tasks in E-athletes, although the results should not be generalized to all executive-function domains.

Sousa et al. [28] assessed physiological and cognitive responses before and after a discrete competitive esports gaming session in university-level players. The study reported increased perceived workload following match play, but did not provide consistent evidence of broad cognitive decline across the assessed tasks. This pattern suggests that short-term competitive gaming may increase psychophysiological load without necessarily producing uniform impairment in cognitive performance. However, the small sample and short observation window limit interpretation of the durability and generalizability of these findings.

Goulart et al. [19] examined nutrition, sleep, physical activity, and cognitive performance in 119 esport athletes using repeated NeuroTracker X assessments and lifestyle measures. The study reported that sufficient protein intake and selected micronutrient adequacy were associated with better cognitive performance, while poorer sleep-related indicators were associated with lower performance. These results indicate that lifestyle factors may be relevant to visual tracking and sustained cognitive performance in esports athletes.

However, the cross-sectional and remote-assessment design limits causal interpretation, and the findings should be viewed as hypothesis-generating rather than as direct evidence that nutritional modification improves cognitive performance.

Taken together, the studies in this domain suggest that acute exercise, perceived workload, nutrition, and sleep-related factors may be relevant to cognitive performance in esports contexts. Nevertheless, the evidence remains limited by small samples, heterogeneous designs, and variation in cognitive measures. Therefore, lifestyle and health-management recommendations for esports players should be framed as promising but preliminary, requiring confirmation through longitudinal, controlled, and representative intervention studies conducted in esports settings.

### Decision-making and training mechanisms

One included qualitative study examined decision-making and training mechanisms in expert League of Legends players and coaches [29]. The study involved five expert players (Mage = 20.20, SD = 2.95) and three coaches (Mage = 24.67, SD = 3.21) from a top European League team. Semi-structured interviews and reflexive thematic analysis were used to explore how decision-making is perceived and trained in high-performance esports contexts. Four interrelated themes were identified: the chronological structure of decisions from pre-game preparation to adaptive in-game responses; the role of experience, intuition, and systematic practice in navigating decision-making processes; the importance of fine-motor execution as the endpoint of cognitive and tactical decisions; and the dynamic interaction between game knowledge and cognitive functions such as working memory and cognitive flexibility. This evidence suggests that decision-making in elite esports is not a single isolated cognitive skill, but a situated process involving game knowledge, time pressure, motor execution, team coordination, and environmental constraints. Because this domain was represented by one qualitative study, the findings should be interpreted as an exploratory framework for future research rather than as definitive evidence of training effects.

## Discussion

This scoping review systematically examined the relationship between esports participation and cognitive function. By synthesizing findings from nine peer-reviewed studies, three core thematic categories were identified: (1) Differences in cognitive function across levels of expertise; (2) Lifestyle, health load, and intervention effects; and (3) Decision-making and training mechanisms. Collectively, these findings indicate that the cognitive performance of esports athletes is influenced not only by their competitive level but also by lifestyle behaviors and the training environment. More broadly, this synthesis provides both theoretical and practical insights for clubs, coaches, and players seeking to identify key factors that shape competitive performance in professional esports.

### Cognitive function and expertise-level differences

The evidence in this domain suggests cognitive differences across expertise levels and game genres, but it does not establish that esports practice itself produces broad cognitive enhancement. Several included studies reported advantages in selected attention, visuospatial, speeded, or sensorimotor outcomes among expert, professional, or long-term players. However, findings were not uniform across all cognitive domains, and evidence for working memory, switching, and inhibition was more mixed. Because most studies were cross-sectional, observed differences may reflect training exposure, self-selection, prior cognitive ability, game-genre demands, or combinations of these factors.

This interpretation is consistent with broader action video-game literature reporting associations with perceptual and attentional skills [7,30], while also requiring caution when applying findings from general gaming populations to competitive esports. Accordingly, esports should be viewed as a promising applied setting for studying domain-specific cognitive performance rather than as a validated model demonstrating generalized cognitive expertise.

### Lifestyle, health load, and intervention effects

The second thematic domain suggests that cognitive performance in esports may be influenced by health behaviors and short-term psychophysiological load, although the current evidence is not sufficient to establish causal mechanisms. Professional, collegiate, and semi-professional players may experience intensive training schedules and prolonged screen exposure, which have been linked in broader esports health research to sleep disturbance, fatigue, and psychological stress [31]. Within the included studies, acute moderate-intensity cycling was associated with short-term improvements in selected Human Benchmark outcomes, including speed accuracy, visual memory, and reaction time [27], whereas a discrete competitive gaming session increased perceived workload without showing consistent cognitive decline [28]. In addition, nutrition and lifestyle data suggested that protein intake, selected micronutrient adequacy, and sleep-related indicators may be associated with visual tracking performance in esport athletes [19]. These findings support the relevance of health-related factors for esports cognition, but they should be interpreted cautiously because the available studies differ in design, sample size, outcome measures, and temporal scope. The broader exercise–cognition literature provides a useful context for interpreting these findings, but direct extrapolation to esports should be made cautiously. Evidence from sport and exercise psychology suggests that aerobic exercise can support executive function and attentional control, potentially through changes in arousal, cerebral blood flow, and neurotrophic processes [32,33]. However, the included esports studies provide only limited direct evidence on these mechanisms. Accordingly, exercise should be discussed as a feasible complementary strategy that may support selected aspects of cognitive readiness and health management in esports players, rather than as a confirmed mechanism for producing broad cognitive enhancement.

From a practical standpoint, these findings suggest that esports training programs should integrate regular recovery, nutrition, and exercise routines into daily schedules, rather than treating them as secondary concerns. A well-regulated physiological foundation not only sustains attention and reaction speed but also reduces the risk of occupational burnout, thereby conferring a tangible competitive advantage. Theoretically, these findings suggest that esports may be a useful applied context for examining how health-related factors are associated with cognitive performance, although the current evidence does not establish causal or neural mechanisms. However, the implementation of these recommendations requires consideration of practical constraints, including training schedules, financial resources, and organizational support within esports teams. Integrating physical exercise programs, nutritional guidance, and cognitive training into existing training routines may require structured time management and institutional support from esports organizations.

### Decision-making and training mechanisms

The decision-making findings should be interpreted within a broader cognitive–motor framework. In the included qualitative study, expert League of Legends players and coaches described decision-making as a dynamic process that begins before the match, continues through rapidly changing in-game situations, and depends on the interaction among game knowledge, working memory, cognitive flexibility, fine-motor execution, and team coordination [29]. This supports the view that esports decision-making is not reducible to a single executive-function component. Instead, it appears to involve the coordination of perceptual information, tactical knowledge, affective regulation, and motor implementation under time pressure.

Recent contextual studies provide complementary perspectives on acute exercise, emotional intelligence and decision-making, esports training, coordination-based activity, cognitive load, and feasible activity-break strategies [34–40]. These studies were not added to the included evidence base because they were either published after the search cut-off, did not focus on competitive esports populations, or addressed broader exercise–cognition mechanisms rather than esports-specific cognitive outcomes. However, they are useful for interpreting the current findings cautiously. For example, research on emotional intelligence and decision-making in esports players suggests that decision-making styles may be shaped by affective and self-regulatory factors, although cross-sectional and self-report designs limit causal interpretation [35]. Similarly, emerging esports training studies using decision-making or neurophysiological outcomes indicate that game-based practice may influence focus or decision-related indicators, but small samples and novice or non-professional participants limit transferability to elite esports contexts [36].

The reviewer-recommended cognitive–motor literature also helps clarify the mechanism implied by the included qualitative evidence. Studies of coordination-based or cognitively engaging physical activities show that motor coordination and inhibitory control may be linked, but effects are task-specific and not uniformly significant across all executive-function measures [37,38]. Research on cognitive load and visual variability further indicates that cognitive task demands and altered visual conditions can interfere with postural or motor performance, supporting the broader concept of cognitive–motor interference under constrained conditions [39]. These findings are relevant to esports because high-level gameplay requires rapid perception–action coupling, allocation of attention under visual complexity, and execution of fine motor responses under time pressure. Nevertheless, these studies should be treated as theoretical support rather than direct evidence that esports training improves cognitive control.

From an applied perspective, the current evidence supports cautious development of decision-specific training strategies, such as role-specific scenario drills, structured review of in-game decision sequences, communication training, and feedback based on representative game situations. However, the empirical basis for these recommendations remains limited. Future studies should test whether such training approaches improve objective decision quality, response timing, team coordination, or transfer to competitive performance using longitudinal or experimental designs.

### Theoretical, practical, and future implications

Taken together, this scoping review suggests that competitive esports may provide a useful applied context for examining cognitive performance under time pressure, visual complexity, and continuous performance feedback. However, the current evidence base does not support strong claims that esports represents a fully validated model of cognitive expertise or that esports participation produces broad cognitive enhancement. Theoretically, the findings point to potentially important links among attentional control, visuospatial processing, decision-making, and fine-motor execution, but these links require confirmation through more rigorous longitudinal and experimental research. Practically, esports organizations and coaches may consider integrating cognitive monitoring, structured recovery, physical activity, and decision-specific training into player-development programs, but such recommendations should be implemented as preliminary, evidence-informed strategies rather than established performance protocols.

Nevertheless, current evidence remains constrained by relatively small sample sizes and the predominance of cross-sectional research designs. Because cross-sectional studies cannot establish temporal or causal relationships, it remains unclear whether enhanced cognitive performance results from intensive esports training or whether individuals with superior baseline cognitive abilities are more likely to succeed in competitive gaming. Future research should therefore employ longitudinal, cross-cultural, and experimental intervention approaches, integrating neuroimaging, behavioral, and physiological indicators to elucidate the dynamic interactions among cognitive function, health load, and performance outcomes. Expanding these frameworks beyond East Asian, European, and North American populations would further clarify how sociocultural contexts shape cognitive training and expertise development in esports. Ultimately, advancing this field requires a multidimensional perspective that integrates neuroscience, sport psychology, and human-performance science to establish a robust, evidence-based foundation for understanding and enhancing cognitive competence in competitive gaming.

Several limitations should be acknowledged. First, only nine studies met the eligibility criteria. Although this number is acceptable for a scoping review in an emerging and narrowly defined field, it limits the robustness, generalizability, and certainty of the conclusions. Second, the included studies were heterogeneous in terms of esports title or genre, participant classification, competitive level, cognitive task, measurement instrument, and statistical reporting. This heterogeneity precluded meta-analysis and required a structured narrative synthesis. Third, most studies used cross-sectional or short-term observational designs, which limits causal inference regarding whether cognitive differences precede esports expertise or emerge through esports training. Fourth, the search was limited to English-language peer-reviewed studies indexed in PubMed and Web of Science, with ScienceDirect used only as a supplementary source for full-text access and reference checking. Relevant studies indexed in other databases or published in other languages may therefore have been missed. Fifth, methodological reporting varied across studies, and the JBI appraisal was used descriptively rather than as a formal risk-of-bias analysis. Future research should use longitudinal, experimental, and cross-cultural designs; adopt standardized cognitive tasks and reporting practices; and include more diverse competitive levels, game genres, and geographical regions.

## Conclusion

This scoping review mapped empirical evidence on the relationship between esports participation and cognitive function in competitive esports populations. The nine included studies suggest that esports expertise and game genre may be associated with domain-specific cognitive profiles, particularly in attention, visuospatial processing, speeded performance, and selected executive-function measures. The evidence also indicates that lifestyle-related factors, short-term physiological load, and decision-making processes may be relevant to cognitive performance in esports contexts.

However, the current evidence base remains small, heterogeneous, and largely non-causal. Most included studies used cross-sectional, short-term, or exploratory designs, and the cognitive tasks and reporting practices varied substantially across studies. Therefore, the findings should not be interpreted as evidence that esports participation broadly improves cognitive function or produces generalized cognitive changes across domains. Instead, they identify promising domains for future research and provide a preliminary framework for examining how cognitive, motor, lifestyle, and performance factors intersect in competitive esports.

Future studies should adopt longitudinal and experimental designs, include more diverse game genres and competitive levels, use standardized cognitive assessments, and report p values, effect sizes, and confidence intervals consistently. Cross-cultural research is also needed to determine whether current findings generalize beyond the limited geographical contexts represented in the existing literature.

## Supporting information

S1 Fig PRISMA-ScR flowchart of record identification, screening, eligibility assessment, and study inclusion. (PDF)

S1 Table Methodological reporting-quality appraisal of the included studies using the Joanna Briggs Institute critical appraisal checklist. (DOCX)

S2 Table Evidence matrix of studies included in the scoping review. The table summarizes study design, participant characteristics, esports title or genre, cognitive domains, cognitive tasks or instruments, main quantitative findings, reported p values, effect sizes or confidence intervals where available, direction of findings, interpretation, and key limitations. NR indicates that the statistic was not reported in the original article.
(DOCX)

## References

[1] Newzoo. Global Esports & Live Streaming Market Report 2022 [Internet]. 2022 [cited 2026 Mar 20]. Available from: https://www.newzoo.com/

[2] Statista. Number of esports viewers worldwide from 2019 to 2025 [Internet]. 2024 [cited 2026 Mar 20]. Available from: https://www.statista.com/

[3] Hamari J, Sjöblom M. What is eSports and why do people watch it? Int Res. 2017;27(2):211–32. doi: 10.1108/IntR-04-2016-0085

[4] Pedraza-Ramirez I, Musculus L, Raab M, Laborde S. Setting the scientific stage for esports psychology. Int Rev Sport Exerc Psychol. 2020;13(1):319–52. doi: 10.1080/1750984X.2020.1723122

[5] Campbell MJ, Toth AJ, Moran AP, Kowal M, Exton C. eSports: A new window on neurocognitive expertise? Prog Brain Res. 2018;240:161–74. doi: 10.1016/bs.pbr.2018.09.006 30390829

[6] Dale G, Joessel A, Bavelier D, Green CS. A new look at the cognitive neuroscience of video game play. Ann N Y Acad Sci. 2020;1464(1):192–203. doi: 10.1111/nyas.14295 31943260

[7] Bediou B, Adams DM, Mayer RE, Tipton E, Green CS, Bavelier D. Meta-analysis of action video game impact on perceptual, attentional, and cognitive skills. Psychol Bull. 2018;144(1):77–110. doi: 10.1037/bul0000130 29172564

[8] Lachowicz M, Żurek A, Jamro D, Serweta-Pawlik A, Żurek G. Changes in concentration performance and alternating attention after short-term virtual reality training in E-athletes: a pilot study. Sci Rep. 2024;14(1):8904. doi: 10.1038/s41598-024-59539-w 38632364 PMC11024211

[9] Pluss MA, Bennett KJM, Novak AR, Panchuk D, Coutts AJ, Fransen J. Esports: The Chess of the 21st Century. Front Psychol. 2019;10:156. doi: 10.3389/fpsyg.2019.00156 30761055 PMC6363684

[10] Glass BD, Maddox WT, Love BC. Real-time strategy game training: emergence of a cognitive flexibility trait. PLoS One. 2013;8(8):e70350. doi: 10.1371/journal.pone.0070350 23950921 PMC3737212

[11] Mancı E, Güdücü Ç, Günay E, Güvendi G, Campbell M, Bediz CŞ. The relationship between esports game genres and cognitive performance: A comparison between first-person shooter vs. multiplayer online battle arena games in younger adults. Entertain Comput. 2024;50:100640. doi: 10.1016/j.entcom.2024.100640

[12] Gong D, Ma W, Liu T, Yan Y, Yao D. Electronic-Sports Experience Related to Functional Enhancement in Central Executive and Default Mode Areas. Neural Plast. 2019;2019:1940123. doi: 10.1155/2019/1940123 30804989 PMC6362490

[13] Carroll JB. Human cognitive abilities: A survey of factor-analytic studies. Cambridge: Cambridge University Press; 1993.

[14] Kowal M, Toth AJ, Exton C, Campbell MJ. Different cognitive abilities displayed by action video gamers and non-gamers. Comput Hum Behav. 2018;88:255–62. doi: 10.1016/j.chb.2018.07.010

[15] Miao H, He H, Hou X, Wang J, Chi L. Cognitive expertise in esport experts: a three-level model meta-analysis. PeerJ. 2024;12:e17857. doi: 10.7717/peerj.17857 39131624 PMC11316462

[16] Bányai F, Griffiths MD, Király O, Demetrovics Z. The Psychology of Esports: A Systematic Literature Review. J Gambl Stud. 2019;35(2):351–65. doi: 10.1007/s10899-018-9763-1 29508260

[17] Nagorsky E, Wiemeyer J. The structure of performance and training in esports. PLoS One. 2020;15(8):e0237584. doi: 10.1371/journal.pone.0237584 32841263 PMC7447068

[18] Nicholson M, Poulus D, Johnson D, Robergs R, Kelly V, McNulty C. Role of a 10-Week Exercise Intervention on Cerebral Hemoglobin Saturation, Cognitive Function, and Heart Rate Variability Within Elite Esports Players: A Pilot Study. J Electron Gaming Esports. 2024;2(1). doi: 10.1123/jege.2024-0007

[19] Goulart JB, Aitken LS, Siddiqui S, Cuevas M, Cardenas J, Beathard KM, et al. Nutrition, lifestyle, and cognitive performance in esport athletes. Front Nutr. 2023;10:1120303. doi: 10.3389/fnut.2023.1120303 37275641 PMC10233150

[20] Munn Z, Peters MDJ, Stern C, Tufanaru C, McArthur A, Aromataris E. Systematic review or scoping review? Guidance for authors when choosing between a systematic or scoping review approach. BMC Med Res Methodol. 2018;18(1):143. doi: 10.1186/s12874-018-0611-x 30453902 PMC6245623

[21] Tricco AC, Lillie E, Zarin W, O’Brien KK, Colquhoun H, Levac D, et al. PRISMA Extension for Scoping Reviews (PRISMA-ScR): Checklist and Explanation. Ann Intern Med. 2018;169(7):467–73. doi: 10.7326/M18-0850 30178033

[22] Joanna Briggs Institute. Critical appraisal tools [Internet]. 2020. Available from: https://jbi.global/critical-appraisal-tools

[23] Benoit JJ, Roudaia E, Johnson T, Love T, Faubert J. The neuropsychological profile of professional action video game players. PeerJ. 2020;8:e10211. doi: 10.7717/peerj.10211 33240605 PMC7678459

[24] Kang JO, Kang KD, Lee JW, Nam JJ, Han DH. Comparison of Psychological and Cognitive Characteristics between Professional Internet Game Players and Professional Baseball Players. Int J Environ Res Public Health. 2020;17(13):4797. doi: 10.3390/ijerph17134797 32635282 PMC7369982

[25] Valls-Serrano C, De Francisco C, Vélez-Coto M, Caracuel A. Visuospatial working memory and attention control make the difference between experts, regulars and non-players of the videogame League of Legends. Front Hum Neurosci. 2022;16:933331. doi: 10.3389/fnhum.2022.933331 35937676 PMC9351611

[26] Lin Z, Jiao F, Huang L, Zhuang J, Liu Y. Enhanced visual attention-based sensorimotor control abilities in long-term first person shooter game players: A gray matter comparison study on different video games. Comput Hum Behav. 2025;166:108582. doi: 10.1016/j.chb.2025.108582

[27] Zhang W, Wang X, Li X, Yan H, Song Y, Li X, et al. Effects of acute moderate-intensity aerobic exercise on cognitive function in E-athletes: A randomized controlled trial. Medicine (Baltimore). 2023;102(40):e35108. doi: 10.1097/MD.0000000000035108 37800783 PMC10553036

[28] Sousa A, Ahmad SL, Hassan T, Yuen K, Douris P, Zwibel H, et al. Physiological and Cognitive Functions Following a Discrete Session of Competitive Esports Gaming. Front Psychol. 2020;11:1030. doi: 10.3389/fpsyg.2020.01030 32547452 PMC7272664

[29] Pedraza-Ramirez I, Musculus L, Raab M, Ramaker B, Laborde S. Zooming in on decision making in esports: Exploring the perceptions of expert players and coaches. Sport Exerc Perform Psychol. 2025;14(2):335–51. doi: 10.1037/spy0000382

[30] Green CS, Bavelier D. Learning, attentional control, and action video games. Curr Biol. 2012;22(6):R197-206. doi: 10.1016/j.cub.2012.02.012 22440805 PMC3461277

[31] Smith M, Sharpe B, Arumuham A, Birch P. Examining the Predictors of Mental Ill Health in Esport Competitors. Healthcare (Basel). 2022;10(4):626. doi: 10.3390/healthcare10040626 35455804 PMC9027719

[32] Hillman CH, Erickson KI, Kramer AF. Be smart, exercise your heart: exercise effects on brain and cognition. Nat Rev Neurosci. 2008;9(1):58–65. doi: 10.1038/nrn2298 18094706

[33] Erickson KI, Voss MW, Prakash RS, Basak C, Szabo A, Chaddock L, et al. Exercise training increases size of hippocampus and improves memory. Proc Natl Acad Sci U S A. 2011;108(7):3017–22. doi: 10.1073/pnas.1015950108 21282661 PMC3041121

[34] Mancı E, Gençtürk U, Günay E, Güdücü Ç, Herold F, Bediz CŞ. The influence of acute sprint exercise on cognition, gaming performance, and cortical hemodynamics in esports players and age-matched controls. Curr Psychol. 2024;43:19643–54. doi: 10.1007/s12144-024-05750-x

[35] Temel V, Aydin H. E-Sports players: emotional intelligence and decision making levels. BMC Sports Sci Med Rehabil. 2025;17(1):330. doi: 10.1186/s13102-025-01388-9 41219941 PMC12607108

[36] Imanian M, Khatibi A, Dhamala M, Moheb E, Heydarinejad S, Veisia E, et al. Verify the effects of esports on cognitive skill: focusing on decision making. BMC Sports Sci Med Rehabil. 2025;17(1):195. doi: 10.1186/s13102-025-01236-w 40646661 PMC12247360

[37] Ltifi MA, Cherni Y, Panaet EA, Alexe CI, Ben Saad H, Vulpe AM, et al. Mini-Trampoline Training Enhances Executive Functions and Motor Skills in Preschoolers. Children (Basel). 2025;12(10):1405. doi: 10.3390/children12101405 41153587 PMC12563777

[38] Başarır B, Canlı U, Şendil AM, Alexe CI, Tomozei RA, Alexe DI, et al. Effects of coordination-based training on preschool children’s physical fitness, motor competence and inhibition control. BMC Pediatr. 2025;25(1):539. doi: 10.1186/s12887-025-05897-x 40629304 PMC12235774

[39] Jouira G, Alexe DI, Moraru CE, Rekik G, Alexe CI, Marinău MA, et al. The influence of cognitive load and vision variability on postural balance in adolescents with intellectual disabilities. Front Neurol. 2024;15:1385286. doi: 10.3389/fneur.2024.1385286 38882695 PMC11176554

[40] Alexe DI, Saha S, Choudhary PK, Alexe CI, Choudhary S, Tohănean DI. Exercise Snacks as a Strategy to Interrupt Sedentary Behavior: A Systematic Review of Health Outcomes and Feasibility. Healthcare (Basel). 2025;13(24):3216. doi: 10.3390/healthcare13243216 41464286 PMC12732512

