# Supplementary material for: The relationship between esports and cognitive function: A scoping review
Source: PLoS One. 2026 Jul 10;21(7):e0352875. doi: 10.1371/journal.pone.0352875 (PMC13353933; doi:10.1371/journal.pone.0352875)

S1 Fig. PRISMA-ScR flowchart of record identification, screening, eligibility assessment, and study inclusion.

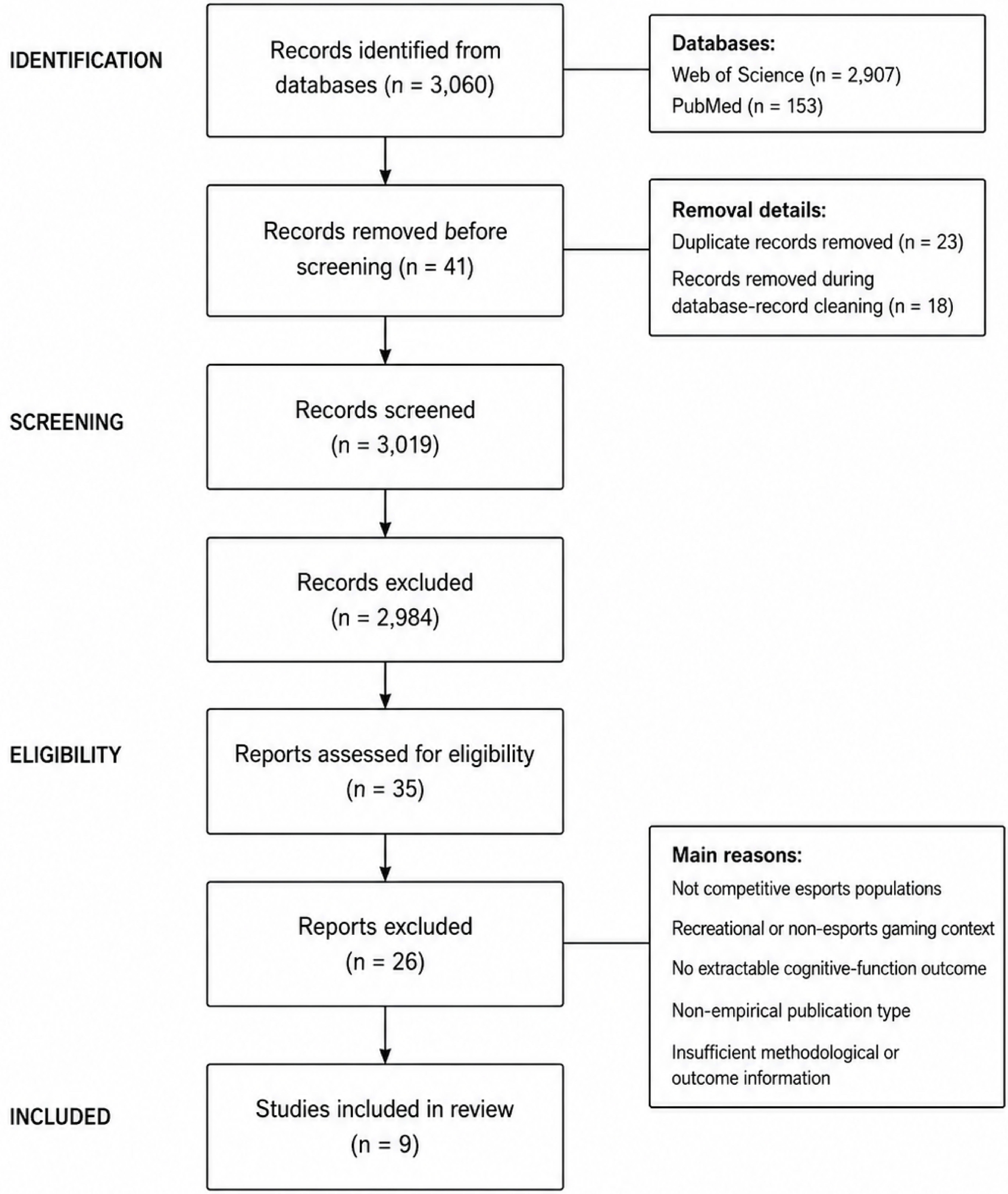

Supplement: S1 Fig — (PDF) [file pone.0352875.s001.pdf]
