# Supplementary material for: The relationship between esports and cognitive function: A scoping review
Source: PLoS One. 2026 Jul 10;21(7):e0352875. doi: 10.1371/journal.pone.0352875 (PMC13353933; doi:10.1371/journal.pone.0352875)
Supplement: S1 Table — (DOCX) [file pone.0352875.s002.docx]

**S1 Table. Methodological reporting-quality appraisal of included studies using the Joanna Briggs Institute (JBI) critical appraisal checklist.**

| **Reference** | **Q1** | **Q2** | **Q3** | **Q4** | **Q5** | **Q6** | **Q7** | **Q8** | **Final score** | **Descriptive interpretation** |
| --- | --- | --- | --- | --- | --- | --- | --- | --- | --- | --- |
| Valls-Serrano et al. (2022) | Yes | Yes | Yes | Yes | Yes | Unclear | Yes | Yes | 7 | Adequate reporting |
| Zhang et al. (2023) | Yes | Yes | Yes | Yes | Unclear | Yes | Yes | Yes | 7 | Adequate reporting |
| Benoit et al. (2020) | Yes | Yes | Yes | Yes | Yes | Yes | Yes | Yes | 8 | Adequate reporting |
| Goulart et al. (2023) | Yes | Yes | Yes | Yes | Yes | Unclear | Yes | Yes | 7 | Adequate reporting |
| Kang et al. (2020) | Yes | Yes | Yes | Yes | Yes | Unclear | Yes | Yes | 7 | Adequate reporting |
| Lin et al. (2025) | Yes | Yes | Yes | Yes | Yes | Unclear | Yes | Yes | 7 | Adequate reporting |
| Mancı et al. (2024) | Yes | Yes | Yes | Yes | Yes | Unclear | Yes | Yes | 7 | Adequate reporting |
| Pedraza-Ramirez et al. (2025) | Yes | Yes | Yes | Yes | Yes | Yes | Yes | Yes | 8 | Adequate reporting |
| Sousa et al. (2020) | Yes | Yes | Yes | Yes | Yes | Unclear | Yes | Yes | 7 | Adequate reporting |

**Note.** Q1–Q8 represent the JBI checklist items applied to describe methodological reporting quality across the included studies. Responses are presented as Yes, No, Unclear, or Not applicable where relevant. Final scores are reported descriptively and should not be interpreted as formal risk-of-bias ratings or certainty-of-evidence grades. The appraisal was not used as an exclusion criterion; instead, it was used to contextualize the transparency and completeness of reporting in the included evidence base.
