# Supplementary material for: The relationship between esports and cognitive function: A scoping review
Source: PLoS One. 2026 Jul 10;21(7):e0352875. doi: 10.1371/journal.pone.0352875 (PMC13353933; doi:10.1371/journal.pone.0352875)
Supplement: S2 Table — The table summarizes study design, participant characteristics, esports title or genre, cognitive domains, cognitive tasks or instruments, main quantitative findings, reported p values, effect sizes or confidence intervals where available, direction of findings, interpretation, and key limitations. NR indicates that the statistic was not reported in the original article. (DOCX) [file pone.0352875.s003.docx]

**S2 Table. Evidence matrix of studies included in the scoping review.**

Note. Significant statistical findings reported in the original studies are shown in bold. NR = not reported in the original article; FPS = first-person shooter; MOBA = multiplayer online battle arena; RT = reaction time; ToL = Tower of London; 3D-MOT = three-dimensional multiple-object tracking; NTx = NeuroTracker X; GM = gray matter; CI = confidence interval.

| **Domain** | **No.** | **Study (year)** | **Design and sample** | **Esports context** | **Cognitive domain(s) and measure(s)** | **Reported statistical evidence** | **Mapped finding** | **Key limitation for interpretation** |
| --- | --- | --- | --- | --- | --- | --- | --- | --- |
| Expertise-level differences | 1 | Valls-Serrano et al. (2022) | Cross-sectional comparison; n = 80 (expert players = 20; regular players = 30; non-videogame players = 30). | League of Legends (MOBA). | Visuospatial working memory (CORSI Block-Tapping); attentional control (Antisaccade Task); switching (Number–Letter Task); inhibition (Stop-Signal Task). | **CORSI block span: H = 7.288, p = .026, Cohen’s d = 0.54; CORSI correct trials: H = 12.322, p = .002, d = 0.78; Antisaccade accuracy: H = 7.896, p = .019, d = 0.58; Antisaccade RT: H = 16.839, p < .001, d = 0.98.** No significant group differences for switching or inhibition (p > .05). | Expert players showed better visuospatial working memory than comparison groups, and players showed attentional-control advantages over non-players. Evidence did not support consistent advantages in switching or inhibition. | Cross-sectional design; expertise and training-selection effects cannot be separated; limited to one MOBA title. |
| Expertise-level differences | 2 | Benoit et al. (2020) | Cross-sectional comparison plus 3D-MOT training; n = 30 (professional Overwatch players = 14; amateur FPS players = 16). | Overwatch / FPS action video-game context. | Processing speed, selective and sustained attention, visuospatial memory, auditory working memory, executive function, manual dexterity, and 3D-MOT thresholds. | **d2 total processed: t(28) = 2.41, p = .02, Hedges’ g = 0.85; d2 TN-E: t(28) = 2.30, p = .03, g = 0.81; d2 fluctuation rate: t(28) = −2.14, p = .04, g = −0.75; Spatial Span total: t(25.5) = 3.52, p < .001, g = 1.27; Spatial Span Forward: t(28) = 3.85, p = .001, g = 1.36; Digit Span total: t(23) = 2.20, p = .04, g = 0.82; 3D-MOT group effect: F(1,24.78) = 5.54, p = .03, β = 0.28, 95% CI [0.05, 0.52].** 3D-MOT learning-rate interaction: p = .28; multivariate discriminant model: p = .11. | Professional players showed advantages in selected attention, visuospatial memory, auditory working memory, and 3D-MOT threshold measures. There was no evidence that professionals improved faster across 3D-MOT training sessions. | Small samples; cross-sectional group definition; uncorrected univariate comparisons; no causal inference about training effects. |
| Expertise-level differences | 3 | Kang et al. (2020) | Cross-sectional comparison; n = 172 (professional esports players = 55; professional baseball players = 57; healthy comparison subjects = 60). | Professional internet gaming; specific game titles not fully specified in the extracted table. | Working memory/problem solving (modified ToL); emotional perception; mental rotation; psychological inventories. | **ToL RT: F = 9.31, p < .01, η = 0.823; Mental Rotation correct rate: F = 19.63, p < .01, η = 0.907; Mental Rotation RT: F = 9.67, p < .01, η = 0.829; Emotional Perception RT: F = 8.62, p < .01, η = 0.812.** ToL correct rate: p = .42; Emotional Perception correct rate: p = .06. | Esports players showed faster ToL performance and higher Mental Rotation accuracy than comparison groups, whereas baseball players showed faster Emotional Perception and Mental Rotation RT. Cognitive profiles differed by sport context rather than showing a uniform esports advantage. | Cross-sectional; esports titles and role-specific expertise are not fully differentiated; cognitive tasks are not game-specific. |
| Expertise-level differences | 4 | Mancı et al. (2024) | Cross-sectional comparison; n = 39 (MOBA players = 17; FPS players = 22). | League of Legends (MOBA) and Valorant (FPS). | Visual working memory/spatial attention (Change Detection); sustained attention/vigilance (Mackworth Clock); visual-spatial/time perception (Timewall); inhibitory control (Flanker). | Change Detection accuracy: F(1,37) = 2.79, p = .103, η² = 0.070; Change Detection RT: p = .76; Timewall accuracy: F(1,36) = 0.55, p = .81, η² = 0.020. **Mackworth correct answers: F(1,37) = 5.82, p = .02, η² = 0.136; Flanker correct responses: F(1,37) = 5.37, p = .02, η² = 0.127; Flanker RT: F(1,37) = 6.18, p = .01, η² = 0.143.** Mackworth RT: p = .30. | FPS players outperformed MOBA players on sustained attention and Flanker-related inhibition/RT measures, while no genre differences were observed for Change Detection or Timewall outcomes. | Small and unequal genre groups; cross-sectional genre comparison; cannot distinguish selection effects from training-related adaptation. |
| Expertise-level differences | 5 | Lin et al. (2025) | Cross-sectional behavioral and structural MRI comparison; n = 108 male participants (FPS = 36; MOBA = 36; controls = 36). | Long-term FPS and MOBA players; controls without video-game experience. | Esports performance tasks related to aiming, visual attention, press reaction, and sensorimotor control; VBM/DBM measures of GM structure. | **Exact Aiming score: F(2,105) = 65.495, P < .001, η² = 0.555; FPS > MOBA, P = .002. Exact Aiming accuracy: F(2,105) = 55.586, P < .001, η² = 0.514; FPS > MOBA, P = .004. Flick Aiming score: F(2,105) = 39.536, P < .001, η² = 0.430; FPS > MOBA, P = .005. Flick Aiming accuracy: F(2,105) = 30.152, P < .001, η² = 0.365. Press Reaction mean RT: P = .002, η² = 0.148; Press Reaction SD RT: F(2,105) = 19.290, P < .001, η² = 0.269; FPS lower SD RT than MOBA, P = .008.** GM differences were reported at q < .05 FDR-corrected. | FPS players showed stronger performance than MOBA players and controls on selected visual attention/sensorimotor tasks and showed structural differences in attention-control, sensorimotor, visual, and related brain regions. | All-male sample; cross-sectional neuroimaging design; GM differences are associations and should not be interpreted as evidence of training-induced neuroplasticity. |
| Lifestyle, health load, and intervention effects | 6 | Zhang et al. (2023) | Randomized crossover trial; n = 34 E-athletes. | Mixed esports / E-athlete context. | Human Benchmark tasks: speed accuracy, visual memory, reaction time, sequence memory, and chimp test after 30 min moderate-intensity cycling (64–76% maximum HR) versus control. | **Immediate post-exercise: speed accuracy, P < .001, Cohen’s d = 1.406, 95% CI [0.717, 2.072]; visual memory, P < .001, d = 1.416, 95% CI [0.725, 2.086]; reaction time, P < .001, d = 1.265, 95% CI [0.610, 1.898]. Thirty minutes post-exercise: speed accuracy, P = .005, d = 0.782, 95% CI [0.227, 1.319]; visual memory, P = .015, d = 0.662, 95% CI [0.127, 1.181]; reaction time, P < .001, d = 0.979, 95% CI [0.386, 1.551].** Sequence memory and chimp test: not significant (p > .05). | Acute moderate-intensity cycling was associated with short-term improvements in selected speeded and visual cognitive tasks, but not in all Human Benchmark outcomes. | Acute laboratory-style intervention; short follow-up; Human Benchmark outcomes may not represent all executive-function domains or in-game performance. |
| Lifestyle, health load, and intervention effects | 7 | Sousa et al. (2020) | Prospective pre–post observational cohort; n = 17 male university esports team players (Overwatch/FPS = 9; League of Legends/MOBA = 8). | Competitive collegiate esports session lasting approximately 151 min on average. | Physiological measures, finger tapping, Trail Making Test, Tower of London, and Stroop task before and after a competitive gaming session. | **Systolic BP increased for FPS gameplay: p = .019; resting-to-peak HR change differed by game type: p = .043; dominant-hand finger tapping improved: F = 11.68, p = .004; TMT-B speed improved: F = 8.32, p = .011; ToL speed improved: F = 7.79, p = .014; Stroop speed improved: F = 5.89, p = .028; Stroop accuracy decreased: F = 4.95, p = .042.** TMT-A: p = .67; ToL accuracy: p = .73; non-dominant finger tapping: p = .95. | After a discrete competitive gaming session, players showed faster performance on selected speeded tasks but reduced Stroop accuracy, suggesting increased speed with possible accuracy or inhibitory-control trade-offs. | Small all-male sample; no non-gaming control condition; pre–post design cannot separate practice, fatigue, arousal, and game-type effects. |
| Lifestyle, health load, and intervention effects | 8 | Goulart et al. (2023) | Cross-sectional remote assessment; n = 119 esport athletes with complete datasets (103 male, 16 female). | Mixed esport athletes; participants classified as professional, elite, or avid players. | Nutrition records, sleep and physical activity indicators, and NeuroTracker X 3D-MOT cognitive-performance sessions. | **Total vegetable intake and average NTx score: r = .272, p = .003. Final sustain session and Stanford Sleepiness Scale: r = −.230, p = .015. Protein intake group difference across 18 core NTx sessions: p = .018, observed power β = 0.812.** Several micronutrients showed significant positive associations with average NTx score (p < .05). | Protein adequacy, selected micronutrient intake, vegetable intake, and lower sleepiness were associated with better NeuroTracker-related performance indicators. | Cross-sectional and remote design; dietary self-report; associations cannot establish that nutrition or sleep modification improves cognition. |
| Decision-making and training mechanisms | 9 | Pedraza-Ramirez et al. (2025) | Qualitative interview study; n = 8 (expert League of Legends players = 5; coaches = 3). | Elite League of Legends team context. | Decision-making processes, game knowledge, cognitive functions, fine-motor actions, and training perceptions assessed through semi-structured interviews and reflexive thematic analysis. | NR for p values/effect sizes because the study used qualitative reflexive thematic analysis. Four themes were reported: chronological structure; navigating the decision-making process; individual fine-motor actions; and the dynamic interplay between game knowledge and cognitive functions. | Decision-making was mapped as a situated process involving pre-game preparation, adaptive in-game responses, experience, intuition, systematic practice, fine-motor execution, game knowledge, working memory, and cognitive flexibility. | Qualitative design; small expert sample from one team context; findings provide an exploratory framework rather than quantitative evidence of training effects. |
